# Cardiac Peroxisome Proliferator-Activated Receptor δ (PPARδ) as a New Target for Increased Contractility without Altering Heart Rate

**DOI:** 10.1371/journal.pone.0064229

**Published:** 2013-05-28

**Authors:** Zhih-Cherng Chen, Kung Shing Lee, Li-Jen Chen, Lin-Yu Wang, Ho-Shan Niu, Juei-Tang Cheng

**Affiliations:** 1 Department of Cardiology, Chi-Mei Medical Center, Yong Kang, Tainan City, Taiwan; 2 Department of Medical Research, Chi-Mei Medical Center, Yong Kang, Tainan City, Taiwan; 3 Department of Surgery, Kaohsiung Municipal Hsiao-Kang Hospital, and Kaohsiung Medical University, Kaohsiung City, Taiwan; 4 Institute of Basic Medical Sciences, College of Medicine, National Cheng Kung University, Tainan City, Taiwan; 5 Department of Pediatrics, Chi-Mei Medical Center, Yong Kang, Tainan City, Taiwan; 6 Department of Nursing, Tzu Chi College of Technology, Hualien City, Taiwan; 7 Department of Pharmacy, Chia Nan University of Pharmacy & Science, Jean-Tae, Tainan City, Taiwan; University of Padua, Italy

## Abstract

**Background and Aims:**

Agents having a positive inotropic effect on the heart are widely used for the treatment of heart failure. However, these agents have the side effect of altering heart rate. It has been established that peroxisome proliferator-activated receptor δ (PPARδ) is mediated in cardiac contraction, however the effect on heart rate is unknown. Thus, we used an agonist of PPARδ, GW0742, to investigate this issue in the present study.

**Methods and Results:**

We used isolated hearts in Langendorff apparatus and hemodynamic analysis in catheterized rats to measure the actions of GW0742 *extra-vivo* and *in vivo*. In diabetic rats with heart failure, GW0742 at a dose sufficient to activate PPARδ reversed cardiac contraction without changes in heart rate. In normal rats, PPARδ enhanced cardiac contractility and hemodynamic dP/dt_max_ significantly more than dobutamine. Both actions were diminished by GSK0660 at a dose enough to block PPARδ. However, GW0742 at the same dose failed to modify heart rate, although it did produce a mild increase in blood pressure. Detection of intracellular calcium level and Western blotting analysis showed that the intracellular calcium concentration and troponin I phosphorylation were both enhanced by GW0742.

**Conclusion:**

Activation of PPARδ by GW0742 increases cardiac contractility but not heart rate. Thus, PPARδ may be a suitable target for the development of inotropic agents to treat heart failure without changing heart rate.

## Introduction

Cardiac glycosides have been used for nearly 200 years, with digitalis being the most commonly used agent [1], [2]. The use of catecholamines is limited due to the short half-life and the fact that they cannot taken orally [3], although dopamine and dobutamine are used in clinic [4]–[9]. Many agents displaying an inotropic effect have been investigated for the treatment of heart failure [10], [11]. However, a concern with most of these agents is a change in heart rate leading to arrhythmia.

Peroxisome proliferator-activated receptors (PPARs) are ligand-activated transcriptional factors that regulate the expression of genes involved in lipid metabolism and inflammation [12]. Three subtypes of PPARs, PPARα, PPARγ, and PPARδ, have been shown to modulate the expressions of various genes to exert bioactivity [12]. PPARα is relatively abundant in tissues with a high oxidative capacity, such as the liver and heart, whereas PPARγ is confined to a limited number of tissues, primarily adipose tissue [12], [13]. The ubiquitously expressed PPARδ enhances lipid catabolism in adipose tissue and muscles [12], and PPARδ-dependent maintenance of inotropic function is crucial for cardiomyocytes [14]–[16]. Deletion of cardiac PPARδ, is accompanied by decreased contraction, increased left ventricular end-diastolic pressure and lowered cardiac output, and leads to decreased contraction and increased incidence of cardiac failure [14]. Previously, we identified that cardiac agents, such as digoxin and dobutamine, can restore cardiac contractility in diabetic rats through an increase in PPARδ expression [17]–[19]. However, the effect of PPARδ on heart rate is still unclear.

GW0742 is a ligand of PPARs, which has been shown to have a 300–1,000-fold selectivity for PPARδ versus the other PPARs [20], and full PPARδ agonist-like actions in cell cultures and animal models [21]–[23]. Thus, we used GW0742 to activate PPARδ in this study in isolated hearts and animals. The main aim of the present study was to clarify that whether the activation of PPARδ increases heart rate in addition to the higher of cardiac contractility.

## Methods

### Materials

Dobutamine (a β_1_ receptor agonist of the sympathetic nervous system) and atenolol (a β-blocker of the sympathetic nervous system) were obtained from Sigma-Aldrich Co. (St Louis, MO, USA). GW0742 (a specific PPARδ agonist) and GSK0660 (a specific PPARδ antagonist) were purchased from Santa Cruz Biotechnology, Inc. (Santa Cruz, CA, USA). The fluorescent probe, fura-2, was purchased from Molecular Probes (Eugene, OR, USA), and antibodies to cardiac troponin I (TnI) and phospho-troponin I (p-TnI) (Ser 23/24) were purchased from Cell Signaling Technology (Beverly, MA, USA).

### Animals

Male Wistar rats, weighing from 200 to 250 g, were obtained from the Animal Center of National Cheng Kung University Medical College. All experiments were performed under anesthesia with 3% isoflurane and all efforts were made to minimize suffering. The animal experiments were approved and conducted in accordance with local institutional guidelines for the care and use of laboratory animals in Chi-Mei Medical Center (No. 100052307) and conformed with the Guide for the Care and Use of Laboratory Animals (Kilkenny et al. Improving bioscience research reporting: the ARRIVE guidelines for reporting animal research. PLoS Biol 2010, Jun 29; 8(6):e1000412), as well as the guidelines of the Animal Welfare Act.

### Streptozotocin (STZ) Induced Type 1-like Diabetic Rats

Male Wistar rats, aged 6 weeks were obtained from the Animal Center of National Cheng Kung University Medical College. Diabetic rats were induced by intravenously (i.v.) injecting STZ at 65 mg/kg (Sigma-Aldrich Inc., USA) into fasting rats as described previously [24]. The animals were considered to be diabetic if they had a plasma glucose concentration over 350 mg/dl.

### Langendorff Apparatus for Isolated Heart Determination

The experiment was performed according to a previous description [25]. The rats were sacrificed under anesthesia with 3% isoflurane and their hearts were excised rapidly and rinsed by immersion in ice-cold Krebs-Henseleit buffer (KHB) (mM: NaCl 118.5, KCl 4.7, MgSO4 1.2, CaCl2 1.8, NaHCO3 25.0, and glucose 11.0 at pH 7.35). The hearts were mounted in the Langendorff apparatus and continuously perfused with warm (37°C) and oxygenated (95% O_2_, 5% CO_2_) KHB at a constant pressure of 70 mmHg. The organ chamber temperature was maintained at 37°C during the experiment. A water-filled latex balloon was inserted through an incision in the left atrium into the left ventricle via the mitral valve and adjusted to a left ventricular end-diastolic pressure (LVEDP) of 5–7 mmHg during initial equilibrium. The distal end of the catheter was connected to an iWorx 214 TM data acquisition system (Ladscrib 2.0 software, iWorx Systems, Inc., Dover, NH, USA) via a pressure transducer for continuous recording. Left ventricular systolic function was assessed by recording the left-ventricular developed pressure (LVDP), which was defined as the difference between left-ventricular systolic pressure (LVSP) and LVEDP. Heart rate and coronary flow rate were monitored simultaneously. In each experiment, after allowing 30 minutes for stabilization with perfusion, the test agents dobutamine (10^−6^ mol/L; M), GW0742 (10^−6^ mol/L; M) or GSK0660 (10^−6^ mol/L; M) were added to the KHB for further analysis.

### Catheterization for Hemodynamic dP/dt Measurement

Temporary pacing leads were used for the short-term study and were placed in the right atrium and RV apex. A venogram imaged in 2 different angulations (left anterior oblique 30° and anteroposterior) was obtained to determine the anatomy of the coronary sinus venous system. An LV pacing electrode (IX-214; iWorx Systems, Inc., Dover, NH, USA) was placed either in the free wall region via the lateral or posterior vein or in the anterior region via the great cardiac vein. After femoral artery and venous puncture using the Seldinger technique [26], pressure transducer catheters were inserted into the heart to provide the RV, aortic, mean blood and LV pressures. Pressure catheters and pacing leads were connected to an external pacing computer (iWorx Systems, Inc., Dover, NH, USA) to monitor the heart rate and to acquire hemodynamic signals. The body temperature of the rats was monitored by digital rectal thermometers showing 37.5°C throughout whole procedure. The rats were injected intravenously with dobutamine (3 mg/kg) [27], [28] or GW0742 (5 mg/kg) [29] with/without GSK0660 (3 mg/kg) [30], and the hemodynamic parameters were recorded continuously throughout the whole experiment.

### Cell Cultures

Primary cultures of neonatal rat cardiomyocytes were prepared by the modification of a previously described method [19]. Briefly, under anesthesia with 3% isoflurane, the hearts of 1- to 2-day-old Wistar rats were excised, cut into 1–2 mm pieces and predigested with trypsin to remove red blood cells. The heart tissue was then digested with 0.25% trypsin and 0.05% collagenase. The dissociated cells were placed in uncoated 100 mm dishes and incubated at 37°C in a 5% CO_2_ incubator for at least 1 h to remove the non-myocytic cells. This procedure caused fibroblasts to predominantly attach to the dishes while most of the cardiomyocytes remained in suspension. The cardiomyocyte-enriched population was then collected and counted. The cells were cultured in Dulbecco/Vogt modified Eagle’s minimal essential medium (DMEM) with 1 mmol/L pyruvate, 10% fetal bovine serum, 100 units/mL penicillin, and 100 units/mL streptomycin. Over 95% of the collected cells were characterized as cardiomyocytes on the basis of the sarcomeric myosin content. On the second day, the medium was replaced. After 3 to 4 days in culture, the cells were used in the experiments. Stock solutions of GW0742 and GSK0660 were prepared with DMSO (0.1%). The cells were treated with GW0742 (10^−6^ mol/L; M) for 30 minutes, washed twice with PBS, and removed by trypsinization. The cells were then collected and subjected to a protein expression assay. Additional treatments with GSK0660 (10^−6^ M) [31], were performed for 30 minutes before the GW0742 treatment.

### Measurement of Intracellular Calcium Concentration

The changes in intracellular calcium were detected using the fluorescent probe fura-2-AM [32]. The neonatal rat cardiomyocytes were placed in buffered physiological saline solution (PSS) containing 140 mM NaCl, 5.9 mM KCl, 1.2 mM CaCl_2_, 1.4 mM MgCl_2_, 11.5 mM glucose, 1.8 mM Na_2_HPO_4_, and 10 mM Hepes-Tris, to which was added 5 µM fura-2-AM, and the solution was incubated for 1 h in humidified 5% CO_2_ and 95% air at 37°C. The cells were washed and incubated for an additional 30 minutes in PSS. The neonatal rat cardiomyocytes were inserted into a thermostatic (37°C) cuvette containing 2 mL of calcium-free PSS. After recording the baseline value, GW0742 was added into the cuvette with/without GSK0660 to detect the free intracellular calcium. The fluorescence was continuously recorded using a fluorescence spectrofluorometer (Hitachi F-2000, Tokyo, Japan). Values of [Ca^2+^]i were calculated from the ratio R = F340/F380 by the formula: [Ca^2+^]i = KdB (R − Rmin)/(Rmax − R), where Kd was 225 nM, F fluorescence, and B the ratio of the fluorescence of the free dye to that of the Ca^2+^-bound dye measured at 380 nm. Rmax and Rmin were determined in separate experiments by using GW0742 to equilibrate [Ca^2+^]j with ambient [Ca^2+^] (Rmax), and the addition of 0.1 mM MnCl_2_ and 1 mmol/L EGTA (Rmin). Background autofluorescence was measured in unloaded cells and subtracted from all measurements.

### Western Blotting Analysis

Protein was extracted from tissue homogenates and cell lysates using ice-cold radio-immuno-precipitation assay (RIPA) buffer supplemented with phosphatase and protease inhibitors (50 mmol/L sodium vanadate, 0.5 mM phenylmethylsulphonyl fluoride, 2 mg/mL aprotinin, and 0.5 mg/mL leupeptin). Protein concentrations were determined with a Bio-Rad protein assay (Bio-Rad Laboratories, Inc., Hercules, CA, USA). Total proteins (30 µg) were separated by SDS/polyacrylamide gel electrophoresis (10% acrylamide gel) using a Bio-Rad Mini-Protein II system. The protein was transferred to expanded polyvinylidene difluoride membranes (Pierce, Rockford, IL, USA) with a Bio-Rad Trans-Blot system. After transfer, the membranes were washed with PBS and blocked for 1 h at room temperature with 5% (w/v) skimmed milk powder in PBS. The manufacturer’s instructions were followed for the primary antibody reactions. Blots were incubated overnight at 4°C with an immunoglobulin-G polyclonal rabbit anti-mouse antibody (Affinity BioReagents, Inc., Golden, CO, USA) (1∶500) in 5% (w/v) skimmed milk powder dissolved in PBS/Tween 20 (0.5% by volume) to bind the target protein such as PPARδ. The blots were incubated with goat polyclonal antibody (1∶1000) to bind the actin which served as the internal control. After the removal of the primary antibody, the blots were extensively washed with PBS/Tween 20 and then incubated for 2 h at room temperature with the appropriate peroxidase-conjugated secondary antibody diluted in 5% (w/v) skimmed milk powder and dissolved in PBS/Tween 20. The blots were developed by autoradiography using an ECL-Western blotting system (Amersham International, Buckinghamshire, UK). The immunoblots of cardiac troponin I (28 kDa) and phospho-troponin I (28 kDa) were quantified with a laser densitometer.

### Electrocardiography

The animals were anaesthetized using 3% isoflurane and placed in the supine position. The right arm, left arm and left leg limb leads were then attached. Heart rate, heart rhythm, and the wave forms were recorded by an iWorx 214 data recorder (IX-214; iWorx Systems, Inc., Dover, NH, USA).

### Statistical Analysis

Results were expressed as mean ± SE of each group. Statistical analysis was carried out using ANOVA analysis and Newman-Keuls post-hoc analysis. Statistical significance was set as *p*<0.05.

## Results

### Effect of GW0742 on Cardiac Performance in Anesthetized Diabetic Rats

The cardiac performance (+dP/dt **_max_** and −dP/dt **_max_**) of the control group was similar to previous reports [33]–[36]. Moreover, the cardiac performance and mean blood pressure (MBP) were both significantly decreased in the diabetic rats compared with the controls. Treatment with GW0742 (5 mg/kg) restored the dP/dt_max_ and MBP in the diabetic rats (Table 1).

**Table 1:** Changes in Cardiac Performance of STZ Rats treated with/without GW0742 (7.5 mg/kg).

	−dP/dt _max_ (mmHg/s)	+dP/dt _max_ (mmHg/s)	MBP (mmHg)	HR (Beat/min)
Control	1658±59	2337±103	109±4	361±15
STZ	865±24 ***	1143±84 ***	81±3**	251±19**
STZ+GW	1644±53	2281±116	102±3	262±21**

Values (mean±SE) were obtained from each group of eight rats.

*** *P*<0.05 and ***P<0.01 for values compared to the control.

### Effect of GW0742 on Cardiac Performance in the Isolated Rat Heart

**Figure 1:**
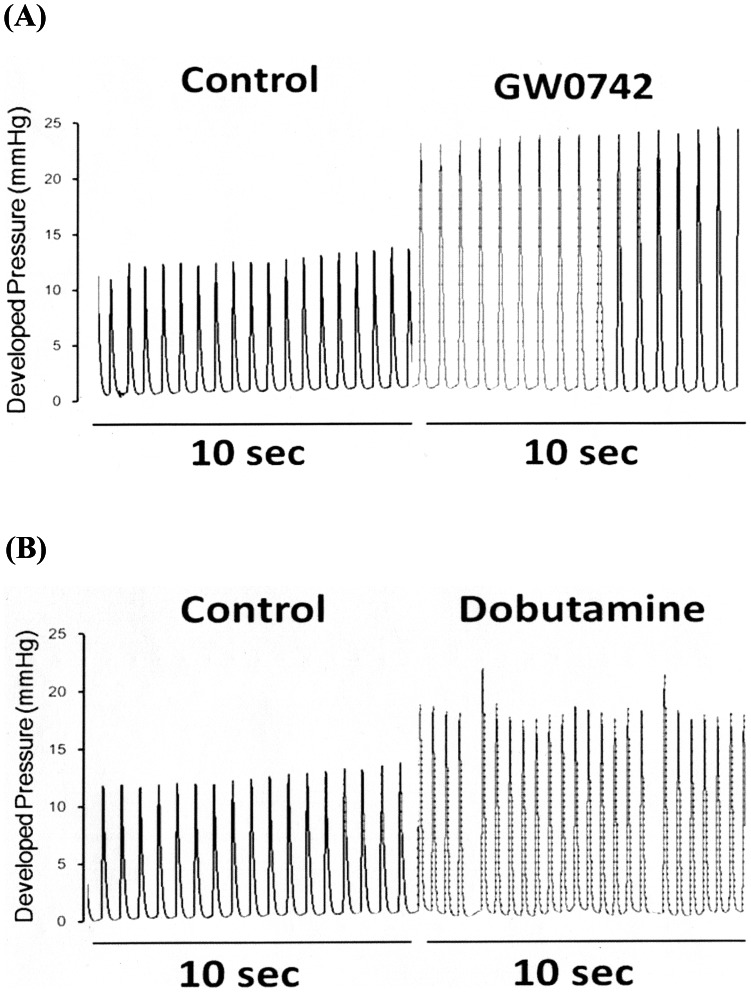
Effects of GW0742 and Dobutamine on Cardiac Performance in Hearts Isolated from Rats. The representative picture shows the change in cardiac performance caused by GW0742 (A) or dobutamine (B) in isolated hearts. Heart rate and cardiac contractility were recorded before and after the perfusion of the test agents.

**Figure 2:**
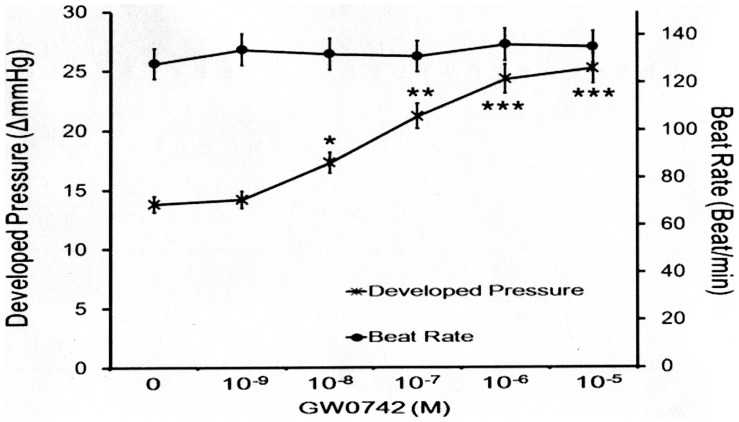
Dose-Related Action of GW0742 Regarding Cardiac Contractility or Heart Rate in Isolated Rat Hearts. Perfusion of GW0742 at various concentrations caused an increase in cardiac contractility but not heart rate. All values are presented as mean ± SEM (n = 8). *P<0.05, **P<0.01 and ***P<0.001 compared with the non-treated controls.

GW0742 at a sufficient dose to activate PPARδ was used to treat the hearts isolated from the rats. The results showed that GW0742 increased the cardiac performance (Fig. 1). We also treated the isolated hearts with dobutamine (10^−6^ M) for comparison (Fig. 1). GW0742 increased cardiac contractility in a concentration-dependent manner (10^−9^–10^−5 ^M) without changes in heart rate (Fig. 2). Furthermore, as shown in Table 2, the cardiac tonic action of GW0742 (10^−6^ M) was diminished by GSK0660 (10^−6^ M). Otherwise, dobutamine increased both cardiac contractility and heart rate at same dose while the increase of cardiac contractility was less significant than GW0742 (Table 2). Atenolol (10^−6^ M) blocked the actions of dobutamine in the isolated hearts but not the action of GW0742 (Table 2).

**Table 2:** Changes of Developed Pressure and Beat Rate in isolated Rat Heart.

	Developed Pressure (ΔmmHg)	Beat Rate (Beat/min)
Control	13.6±0.3	132±8
Dobutamine	21.42±1.4 **	189±12 **
Dobutamine+Atenolol	14.1±0.4	138±11
GW0742	24.45±1.1 ***	144±7
GW0742+ Atenolol	24.23±1.8 ***	141±6
GW+GSK	14.3±0.8	137±11
GSK0660	13.87±0.9	128±8

Isolated rat hearts were treated with GW0742 (10^−6^ M) with/without GSK0660 (10^−6^ M) or with atenolol (10^−6^ M) using Dobutamine (10^−6^ M) with/without atenolol (10^−6^ M) as reference. Values (mean±SE) were obtained from each group of 8 rats.

** *P*<0.01 and ***P<0.01 as compared with the control.

### Effect of GW0742 on Cardiac Performance in the Anesthetized Rats

**Figure 3:**
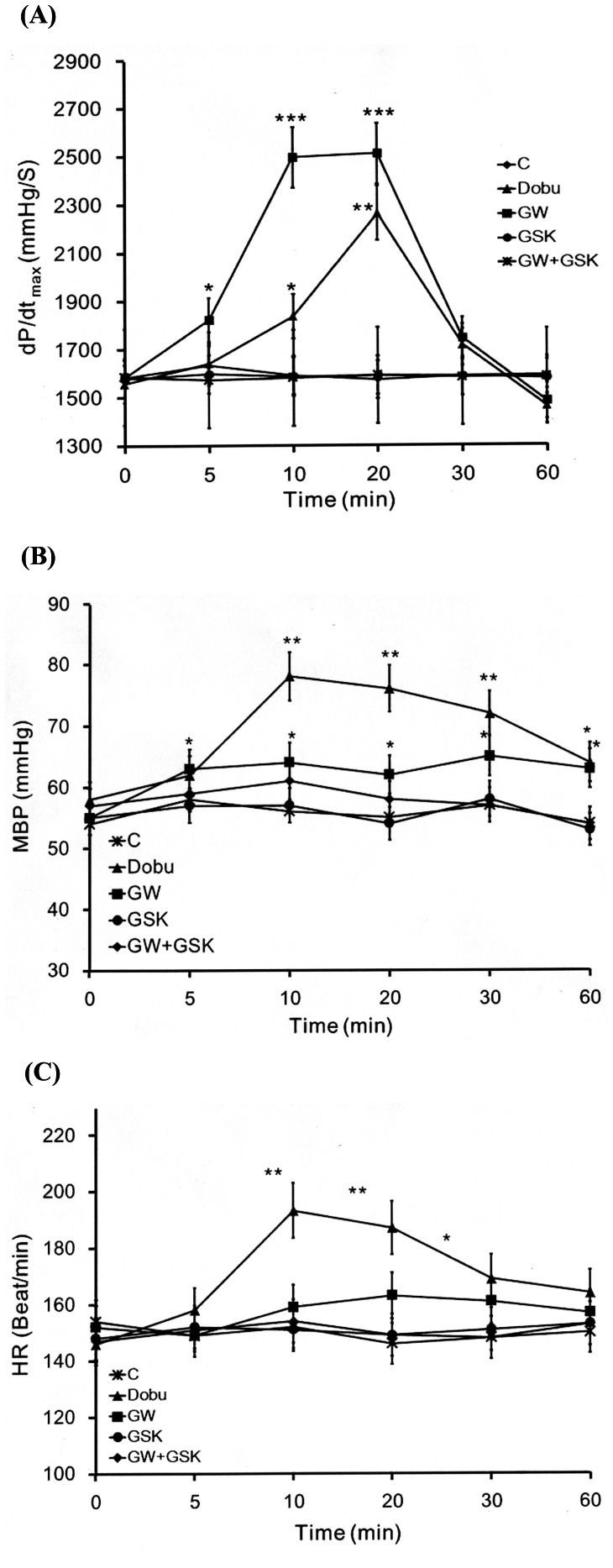
Effects of GW0742 or Dobutamine on Cardiac Performance in Anesthetized Rats. The effects of dobutamine and co-administration of GW0742 and/or GSK0660 were investigated in the anesthetized rats. The changes in hemodynamic dP/dt (A), mean blood pressure (MBP) (B) and heart rate (HR) (C) were recorded continuously throughout the whole experiment. All values are presented as mean ± SEM (n = 8). *P<0.05, **P<0.01 and ***P<0.001 compared with the control group.

The dP/dt_max_ was significantly increased by GW0742 in the anesthetized rats compared with the vehicle-treated controls. However, this effect disappeared in the rats receiving co-administration of GW0742 (5 mg/kg, i.v.) and GSK0660 (3 mg/kg, i.v.) (Fig. 3A). GW0742 seemed to be more effective in increasing cardiac contraction than dobutamine. Treatment of GW0742 did not modify the heart rate (Fig. 3C) but produced a slight increase in blood pressure that was also blocked by GSK0660 (Fig. 3B). This result was markedly different from the actions of dobutamine which significantly increased cardiac contraction in addition to blood pressure and heart rate (Fig. 3).

### Effect of GW0742 on Intracellular Calcium in Neonatal Rat Cardiomyocytes

**Figure 4:**
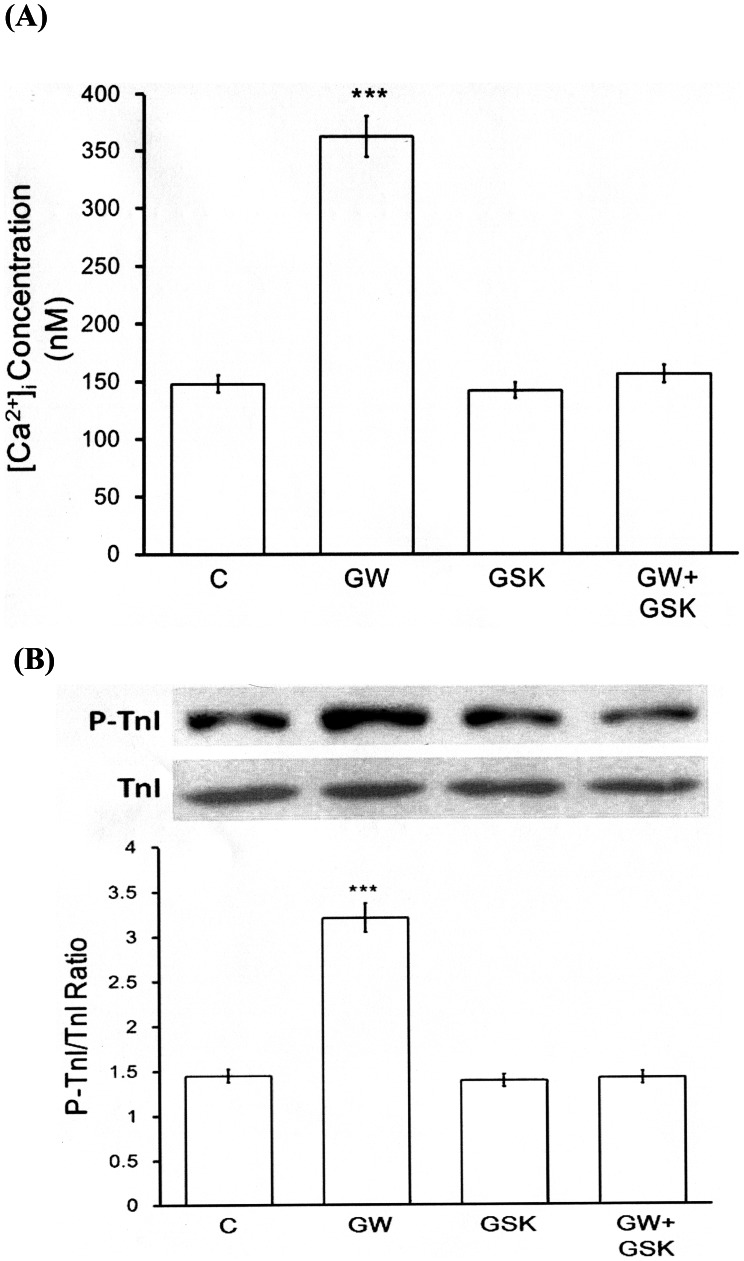
Effects of GW0742 on Intracellular Calcium and TnI Phosphorylation in Neonatal Rat Cardiomyocytes. (A) Changes in intracellular calcium were detected with Fura-2 by using a fluorescence spectrofluorometer. The neonatal rat cardiomyocytes were placed in buffered physiological saline solution with 5 µM of fura-2-AM, and incubated for 1 h. After recording the baseline value, GW0742 was added into the cuvette with/without GSK0660 to detect the free intracellular calcium. (B) Effect of GW0742 on TnI phosphorylation in the neonatal rat cardiomyocytes. Cells were treated with GW0742 for 30 minutes and were then harvested to measure the protein level of TnI phosphorylation by Western blotting analysis. All values are presented as mean ± SEM (n = 8). ***P<0.001 compared with the control group.

The fluorescent probe, fura2-AM, was used to detect changes in intracellular calcium level in the neonatal rat cardiomyocytes, and GW0742 at an effective concentration was found to increase the intracellular calcium level. This effect disappeared in the cardiomyocytes that were co-incubated with GW0742 and GSK0660 (Fig. 4A), however, incubation with GSK0660 alone did not affect the intracellular calcium level in the neonatal rat cardiomyocytes (Fig. 4A).

### Effect of GW0742 on TnI Phosphorylation in Neonatal Rat Cardiomyocytes

The neonatal rat cardiomyocytes were treated with GW0742 to identify changes in TnI phosphorylation, and the level of TnI phosphorylation was found to be markedly raised by GW0742 at an effective concentration (10^−6^ M). In addition, this effect was reversed by GSK0660 at a concentration that did not modify the level of TnI phosphorylation (Fig. 4B).

### Effects of GW0742 on Electrocardiography (ECG) in the Anesthetized Rats

**Figure 5:**
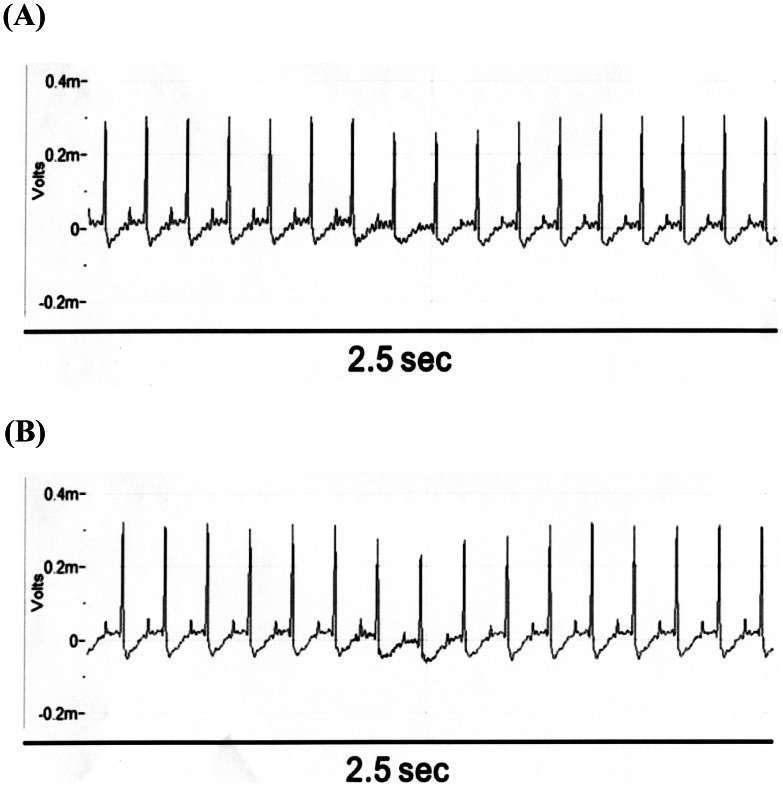
Effect of GW0742 on Electrocardiographic Pattern and Changes. There was no difference between the vehicle treated group (A) and the GW0742 treated group (B).

There were no marked changes in ECG after treatment with GW0742. Moreover, similar QRS intervals were observed in rats receiving GW0742 or vehicle (Fig. 5).

## Discussion

In the present study, we found that activation of PPARδ by the selective agonist GW0742 increased cardiac contractility but not heart rate. In hearts isolated from rats, GW0742 enhanced cardiac contractility in a dose-dependent manner, and this action was diminished by GSK0660 at a concentration sufficient to block PPARδ. The decreased cardiac performance and blood pressure in the type 1-like diabetic rats were both reversed by GW0742 at a dose sufficient to activate PPARδ. In the anesthetized rats, cardiac contraction (dP/dt_max)_ was also significantly increased by GW0742 and this effect was blocked by GSK0660. However, heart rate was not changed by this dose of GW0742. Meanwhile, dobutamine was found to increase cardiac contractility and heart rate in the isolated hearts. In the neonatal rat cardiomyocytes, GW0742 increased cellular calcium levels and troponin phosphorylation directly. Thus, to the best of our knowledge, this is the first study to show that activation of PPARδ may increase cardiac contractility but not heart rate.

It has been established that PPARδ plays an important role in the regulation of cardiac performance [17]–[19]. In the present study, treatment with GW0742 restored the cardiac performance and blood pressure in diabetic rats. Also, we demonstrated that activation of PPARδ using the selective agonist GW0742 enhanced cardiac contractility in the isolated hearts and the hemodynamic dP/dt_max_ in the rats. Both actions of GW0742 were blocked by GSK0660 at a concentration sufficient to block PPARδ [37], [38], which is consistent with previous reports [39].

A change in heart rate is the most serious side effect of cardiac agents [40], [41]. This effect may be due to a direct effect on cardiac β receptors [42] and/or via release of endogenous norepinephrine [43], [44]. In the present study, we demonstrated that dobutamine increased cardiac contractility and heart rate. This action of dobutamine was attenuated by atenolol at a concentration sufficient to block β receptors [45], [46]. Activation of cardiac β receptors by dobutamine can thus be identified, as described previously [47], [48], because activation of cardiac β receptors can result in positive inotropic and chronotropic effects. However, GW0742 enhanced cardiac contractility but not heart rate in isolated heart. In addition, GW0742 generated cardiac tonic action in animals without impacting the heart rate. GW0742 enhanced cardiac contractility without affect heart rate, which may explain why the dP/dt was even higher than that observed in the dobutamine treated group. The increase in cardiac output caused by GW0742 resulted in a mildly higher blood pressure in animals. The effect of dobutamine on cardiac contractility appeared to be transient with a peak after 20 minutes from the administration, while the effect on mean blood pressure appeared to be constant throughout the reported time frame. This may be due to the changes in heart rate were elevated after 10 minute and then continued throughout the reported time frame. However, there was no effect on heart rate and a slight elevation of mean blood pressure in the rats treated with GW0742. Therefore, cardiac performance in response to GW0742 treatment was markedly different than that observed with dobutamine.

Troponin I (TnI) is an inhibitory unit of the troponin complex associated with thin filaments, and it inhibits actomyosin interactions at the diastolic level of intracellular Ca^2+^ [49], [50]. Modulation of myofilament properties by alterations in TnI phosphorylation has been found to have a profound effect on cardiac contractility [51]. Phosphorylation of TnI has been shown to increase the cross-bridge cycling rate, leading to an increase in power output [50], [51]. Ca^2+^ is mainly involved in muscle contraction [49]–[52]. Contraction of cardiac muscles relies upon interactions between ATP and Ca^2+^, both of which must be present in adequate amounts [53]. We observed that GW0742 had the ability to increase the amount of intracellular calcium in cardiomyocytes, and this seemed to be related to the higher cardiac contractility.

It has been showed that TnI phosphorylation most likely acts through an enhanced off rate during Ca^2+^ exchange with contractile protein, leading to an increase in cardiac output [53]–[57]. Consistent with this, we found that TnI phosphorylation was elevated in the neonatal rat cardiomyocytes exposed to GW0742. Thus, direct activation of PPARδ by GW0742 may result in a higher level of TnI phosphorylation. Both changes caused by GW0742 in the cardiomyocytes may explain the increase in cardiac contractility. Moreover, the ECG data showed that GW0742 was not effective in cardiac conduction. A possible explanation for this seems to be related to the absence of PPARδ in the heart conduction system. However, the real mechanism(s) for the lack of effect on heart rate caused by the activation of PPARδ using GW0742 requires further investigations.

According to these findings, we proposed that the activation of PPARδ by its specific agonist increases intracellular calcium release, which then activates calmodulin and/or calcineurin, resulting in cardiac troponin phosphorylation. Subsequently, the cardiac contractility will be enhanced. Taken together, we suggest that PPARδ is a suitable target for the development of cardiac tonic agents that do not alter the heart rate. With such agents, arrhythmia can be ignored for treatment of heart failure.
